# TNFAIP1 contributes to the neurotoxicity induced by Aβ_25–35_ in Neuro2a cells

**DOI:** 10.1186/s12868-016-0286-3

**Published:** 2016-07-18

**Authors:** Ning Liu, Zhanyang Yu, Yu Xun, Miaomiao Li, Xiaoning Peng, Ye Xiao, Xiang Hu, Yi Sun, Manjun Yang, Shiquan Gan, Shishan Yuan, Xiaoying Wang, Shuanglin Xiang, Jian Zhang

**Affiliations:** College of Medicine, Hunan Normal University, Changsha, China; Key Laboratory of Protein Chemistry and Development Biology of State Education Ministry of China, College of Life Sciences, Hunan Normal University, Changsha, 410081 China; Neuroprotection Research Laboratory, Department of Neurology and Radiology, Massachusetts General Hospital, Neuroscience Program, Harvard Medical School, Boston, MA USA; Department of Pathology, The Second Xiangya Hospital of Central South University, Changsha, Hunan China

**Keywords:** TNFAIP1, Amyloid-beta, Alzheimer’s disease, Neurotoxicity, Neuro2a cells

## Abstract

**Background:**

Amyloid-beta (Aβ) accumulation is a hallmark of Alzheimer’s disease (AD) that can lead to neuronal dysfunction and apoptosis. Tumor necrosis factor, alpha-induced protein 1 (TNFAIP1) is an apoptotic protein that was robustly induced in the transgenic *C. elegans* AD brains. However, the roles of TNFAIP1 in AD have not been investigated.

**Results:**

We found TNFAIP1 protein and mRNA levels were dramatically elevated in primary mouse cortical neurons and Neuro2a (N2a) cells exposed to Aβ_25–35_. Knockdown and overexpression of TNFAIP1 significantly attenuated and exacerbated Aβ_25–35_-induced neurotoxicity in N2a cells, respectively. Further studies showed that TNFAIP1 knockdown significantly blocked Aβ_25–35_-induced cleaved caspase 3, whereas TNFAIP1 overexpression enhanced Aβ_25–35_-induced cleaved caspase 3, suggesting that TNFAIP1 plays an important role in Aβ_25–35_-induced neuronal apoptosis. Moreover, we observed that TNFAIP1 was capable of inhibiting the levels of phosphorylated Akt and CREB, and also anti-apoptotic protein Bcl-2. TNFAIP1 overexpression enhanced the inhibitory effect of Aβ_25–35_ on the levels of p-CREB and Bcl-2, while TNFAIP1 knockdown reversed Aβ_25–35_-induced attenuation in the levels of p-CREB and Bcl-2.

**Conclusion:**

These results suggested that TNFAIP1 contributes to Aβ_25–35_-induced neurotoxicity by attenuating Akt/CREB signaling pathway, and Bcl-2 expression.

## Background

Alzheimer’s disease (AD) is a chronic neurodegenerative disease that is characterized by the accumulation of amyloid-beta (Aβ) plaques, neurofibrillary tangles, and neuronal loss in various brain regions [1–3]. The 37–43 amino acid Aβ fragments in the brain are originally derived from the β-amyloid precursor protein (APP) via proteolytic processing by β- and γ-secretase [4]. Aβ_1–40_ and Aβ_1–42_ are two major neurotoxic Aβ fragments, which were mainly generated in the amyloidogenic processing pathway by the actin of β- and γ-secretase [4]. Aβ_25–35_ is not naturally generated in the brain, instead it represents the neurotoxic fragment of Aβ_1–40_ or Aβ_1–42_, and therefore is often used to mimic the neurotoxic role of Aβ_1–40_ or Aβ_1–42_ in experimental studies [5]. Despite increasing evidence showing that Aβ could cause morphological and biochemical characteristics of apoptosis such as chromatin condensation, DNA fragmentation, caspase activation and subsequent activation of apoptotic signaling pathways [6–8], the molecular mechanisms underlying the neurotoxic effect of Aβ have not been fully elucidated. Therefore, identification of key players and mechanisms of Aβ-induced neurotoxicity would benefit the development of novel therapeutic strategies for preventing and treating AD.

Tumor necrosis factor, alpha-induced protein 1 (TNFAIP1) was originally identified as a gene whose expression can be induced by the tumor necrosis factor alpha (TNFα) in umbilical vein endothelial cells [9]. *TNFAIP1* gene is found to be an evolutionarily extremely conserved single-copy gene [9], implying that TNFAIP1 has an important physiological role, which is yet to be explored. TNFAIP1 has been demonstrated to interact directly with proliferating cell nuclear antigen (PCNA) and the small subunit (p50) of DNA polymerase δ, implying that it may be involved in DNA synthesis or DNA repair [10, 11]. Kim et al. [12] found that RhoB induces apoptosis by interacting with TNFAIP1 via a JNK-mediated signaling mechanism, suggesting that TNFAIP1 is an apoptosis-related protein. In addition, the transcription levels of TNFAIP1 had been found to be robustly induced in the transgenic *C. elegans* AD brains and post-mortem AD brain [13, 14], suggesting TNFAIP1 may also involve in the process of AD development. Moreover, a recent study implied that estrogen may affect hippocampal-related diseases by regulating TNFAIP1 [15]. However, the role of TNFAIP1 in AD has not been demonstrated.

In the present study, we examined the roles of TNFAIP1 in Aβ_25–35_-induced apoptosis in neuronal cell line by testing whether the neuronal apoptosis induced by Aβ_25–35_ is associated with the expression of TNFAIP1 protein, and if so, whether apoptosis can be blocked by inhibition of TNFAIP1 expression using TNFAIP1 siRNA. In addition, to further clarify the signal transduction pathways involved in the neurotoxicity induced by Aβ, we also examined the potential signal transduction pathways involved in the apoptosis induced by TNFAIP1.

Our findings demonstrated that TNFAIP1 can be induced by Aβ_25–35_. Overexpression of TNFAIP1 promotes Aβ_25–35_-induced neurotoxicity, whereas knock-down of TNFAIP1 blocks Aβ_25–35_-induced neurotoxicity. In addition, our results suggested that TNFAIP1 induced by Aβ_25–35_ can further inactivate the Akt/CREB signaling pathway, which in turn downregulates Bcl-2 expression.

## Methods

### Cell culture and transfection

Animal experiments were following protocols approved by the Ethic Committee of Hunan Normal University, and the Institutional Animal Care and Use Committee of Massachusetts General Hospital in compliance with the NIH Guide for the Care and Use of Laboratory Animals. Primary mouse cortical neurons were isolated form 15 day embryonic cortex obtained from pregnant C57BL/6 female mouse as described before [16]. The cell pellets were resuspended in neuron basal medium, supplemented with 2 % B27 supplement. Cells were seeded at a density of 3 × 10^5^ cells/mL into 6 cm wells plate pre-coated with poly-d-lysine. Medium was half changed every 4 days. All experiments were performed on cultures at days 7–9 in vitro.

The mouse Neuro2A (N2a) neuroblastoma cell line was obtained from the American type culture collection (ATCC). The N2a cell line was cultured in a humidified (5 % CO_2_, 37 °C) incubator in DMEM supplemented with 10 % heat-inactivated FBS and 50 U/mL penicillin/streptomycin (Invitrogen). The cells were seeded at 1.5 × 10^5^ cells/mL in 24-wells plates or 6-wells plates. For transfection, N2a cells were grown on 24-wells plates to approximately 70 % confluence and then transiently transfected with Control siRNA and TNFAIP1 siRNA (Santa Cruz Biotechnology) or pCMV-myc and pCMV-myc-TNFAIP1 (Myc-TNFAIP1, cloned previously [11]) using Lipotectamine™ 2000 (Invitrogen) following the manufacturer’s protocol.

### Preparation of β-amyloid fragment Aβ_25–35_

Aβ_25–35_ was purchased from Sigma-Aldrich, and dissolved in deionized distilled water at 1 mM and then aged for 5 days in a humid chamber at 37 °C before being added to the culture medium [17]. For treatment of cells, 1 mM of Aβ_25–35_ was further added into the medium at a final concentration of 2, 5, 10 and 20 μM, respectively.

### Cell viability assay

Cell viability was measured by MTT assay according to manufacturer’s protocol. N2a cells were grown on 24-wells plates to approximately 70 % confluence and then transfected with TNFAIP1 siRNA or Myc-TNFAIP1 for 24 h, followed by treatment of Aβ_25–35_ for another 24 h. After treatments, cells were rinsed with PBS and replaced with fresh DMEM containing MTT. The cells were then incubated at 37 °C for 4 h under 5 % CO_2_/95 % air. After the medium was removed, DMSO was added into each well, the absorbance at 570 nm was measured on a microplate reader. Percentages of live cell counts were used for assay normalization.

### Real-time PCR

The RT-PCR primers for mouse TNFAIP1 and 18sRNA were purchased from SABiosciences. Total RNA from N2a cells was isolated with RNeasy Lipid Tissue Mini Kit (Qiagen) according to the manufacturer’s instructions. Then cDNA was synthesized with SuperScript system (Invitrogen). The mRNA levels of TNFAIP1 and 18sRNA were measured by quantitative RT-PCR using SYBR green kit (Applied Biosystems) in an ABI 7000 real-time PCR system (Applied Biosystems). The PCR conditions were as follows: Initial denaturation of DNA, 95 °C for 5 min; denaturation, 32 cycles of 95 °C for 35 s; annealing, 60 °C for 35 s; extension, 72 °C for 35 s; and final extension, 72 °C for 5 min. Data were analyzed according to the comparative threshold cycle method with expression for sample normalization. RT-PCR assay was performed in triplicate for each sample to ensure reproducibility.

### Western blot analysis

Mouse primary cortical neurons or N2a cells were rinsed with PBS, then lysed in cell lysis buffer containing protease inhibitors (Sigma). Equal amounts of total protein (20 μg) were separated using 4–12 % SDS-PAGE gel (Invitrogen) and transferred onto nitrocellulose membrane (Invitrogen). The membrane was blocked with 5 % dry milk in 10 mM PBS buffer (pH 7.2) for 1 h at room temperature. Immunoblots were then performed overnight at 4 °C by incubation with the following primary antibodies: rabbit polyclonal antibodies against CREB (1:2000, Cell Signaling Technology), pCREB (1:1000, Cell Signaling Technology), AKT (1:1000, Cell Signaling Technology), pAKT (1:1000, Cell Signaling Technology), caspase-3 (1:1000, Cell Signaling Technology), cleaved caspase-3 (1:1000, Cell Signaling Technology), TNFAIP1 (1:500, Nanjing Chuanbo Biotech Co, Ltd, China), and mouse monoclonal β-actin antibody (1:5000, sigma), followed by incubation with horseradish peroxidase-conjugated secondary antibodies (1:5000, goat anti-mouse IgG-HRP or goat anti-rabbit IgG-HRP, Abmart) for 1 h at 37 °C. After washed by TTBS, immunolabeling was detected by enhanced chemiluminescence (ECL, Amersham Pharmacia Biotech) according to the manufacturer’s protocol and then exposed to film (X-Omat; Eastman Kodak Co.).

### Statistical analysis

Data were expressed as mean ± SD. Three to five separate experiments were performed. Data were analyzed using ANOVA with Tukey post hoc tests (SPSS version 18.0). Statistical significance was at p < 0.05.

## Results

### Aβ_25–35_ induces TNFAIP1 expression

**Fig. 1:**
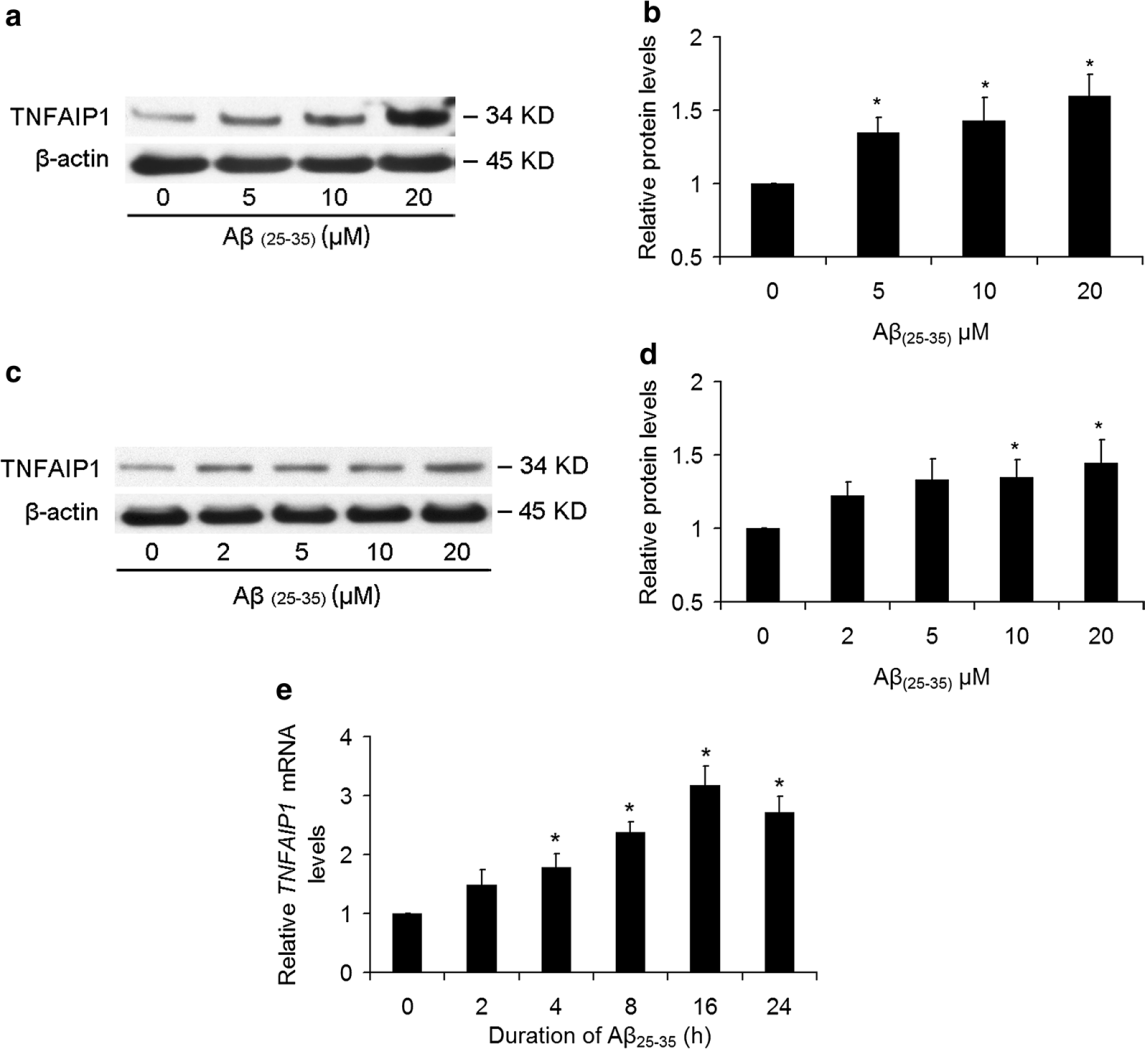
Effect of Aβ_25–35_ on TNFAIP1 expression. **a** TNFAIP1 protein levels were measured by Western blot in mouse primary cortical neurons cultured in 6 wells plate and treated with dose-dependent Aβ_25–35_ (0–20 μM). **b** Quantitative analysis of TNFAIP1 protein levels (fold of control) in mouse primary cortical neurons. Data are expressed as mean ± SD, n = 4, *p < 0.05 compared with Aβ_25–35_ (0 μM) (PBS). **c** TNFAIP1 protein levels were measured by Western blot in N2a cells cultured in 6 wells plate and treated with dose-dependent Aβ_25–35_ (0–20 μM). **d** Quantitative analysis of TNFAIP1 protein levels (fold of control) in N2a cells. Data are expressed as mean ± SD, n = 4, *p < 0.05 compared with Aβ_25–35_ (0 μM) (PBS). **e** TNFAIP1 mRNA levels were determined by RT-PCR in N2a cells cultured in 6 wells plate and treated with 20 μM of Aβ_25–35_. Data are expressed as mean ± SD, n = 3, *p < 0.05 compared with Aβ_25–35_ (0 h)

To determine whether Aβ_25–35_ can increase the TNFAIP1 protein expression, mouse primary cortical neurons were treated with Aβ_25–35_ at different doses for 24 h, and Western blot was used to examine TNFIAP1 protein level. As shown in Fig. 1a, TNFAIP1 expression was increased by Aβ_25–35_ in a dose-dependent manner, and 5, 10 and 20 μM of Aβ_25–35_ significantly upregulated TNFAIP1 protein levels (Fig. 1b). Similar results were obtained in N2a cells (Fig. 1c), as TNFAIP1 protein expression in N2a was also significantly increased by Aβ_25–35_ in a dose-dependent manner (Fig. 1d). Furthermore, the endogenous *TNFAIP1* mRNA levels in N2a cells were detected by Real-time PCR. As expected, Aβ_25–35_ treatment resulted in a significant increase in *TNFAIP1* mRNA levels, and treatment with 20 μM of Aβ_25–35_ for 16 h led to the highest expression of *TNFAIP1* mRNA, but prolonged 24 h treatment did not further increase the *TNFAIP1* mRNA levels (Fig. 1e).

### TNFAIP1 contributes to Aβ_25–35_-induced cell toxicity in N2a cells

**Fig. 2:**
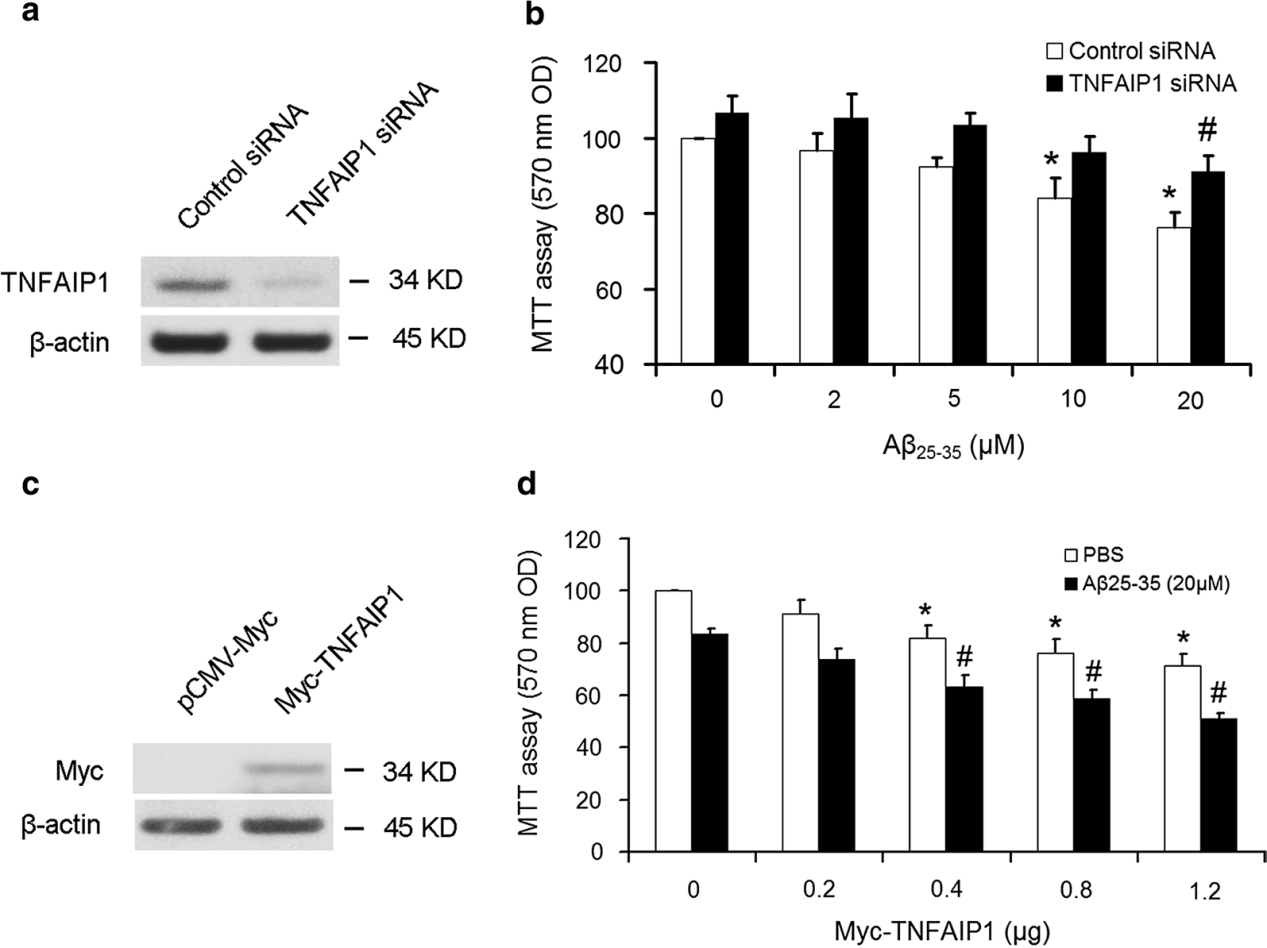
Effect of TNFAIP1 on Aβ_25–35_-induced neuronal cell death. **a** TNFAIP1 protein levels were measured by Western blot in N2a cells cultured in 12 wells plate and transfected with 20 pmol of Control siRNA or TNFAIP1 siRNA. **b** MTT reduction was measured by MTT assay in N2a cells cultured in 12 wells plate and transfected with 20 pmol of Control siRNA or TNFAIP1 siRNA, followed by treated by dose-dependent Aβ_25–35_ (0–20 μM). Data are expressed as mean ± SD, n = 4, *p < 0.05 compared with Aβ_25–35_ (0 μM), ^#^p < 0.05 compared with Control siRNA plus Aβ_25–35_ (20 μM). **c** Myc-TNFAIP1 protein levels were measured by Western blot in N2a cells cultured in 12 wells plate and transfected with 1.2 μg of pCMV-Myc or Myc-TNFAIP1. **d** MTT reduction was measured by MTT assay in N2a cells cultured in 12 wells plate and transfected with dose-dependent Myc-TNFAIP1 (0–1.2 μg), followed by treated by PBS or Aβ_25–35_ (20 μM). Data are expressed as mean ± SD, n = 4, *p < 0.05 compared with Myc-TNFAIP1 (0 μg) plus PBS. ^#^p < 0.05 compared with Myc-TNFAIP1 plus PBS

To further investigate whether TNFAIP1 is involved in Aβ_25–35_-induced cell death, N2a cells were transiently transfected with Control siRNA or TNFAIP1 siRNA for 24 h, and then treated with indicated dose of Aβ_25–35_ for another 24 h. Western blot showed that the endogenous TNFAIP1 protein expression was significantly suppressed by specific TNFAIP1 siRNA but not Control siRNA (Fig. 2a). MTT assay indicated that the cell viability in Control siRNA transfected-N2a cells was significantly decreased when exposed to 10 or 20 μM of Aβ_25–35_. However, inhibition of TNFAIP1 by TNFAIP1 siRNA significantly rescued the viability of N2a cells under 20 μM Aβ_25–35_ (Fig. 2b). Furthermore, to investigate whether TNFAIP1 is associated with increased susceptibility to Aβ_25–35_, the effect of TNFAIP1 overexpression on Aβ_25–35_-reduced the cell viability was also investigated. Transfection of Myc-TNFAIP1 into N2a cells led to significant overexpression of TNFAIP1 protein (Fig. 2c), and Aβ_25–35_-induced cell death was further significantly exacerbated by TNFAIP1 overexpression (Fig. 2d). Taken together, these results indicate that TNFAIP1 contributes to Aβ_25–35_-induced cell toxicity of N2a cells.

### TNFAIP1 contributes to Aβ_25–35_-induced apoptosis

**Fig. 3:**
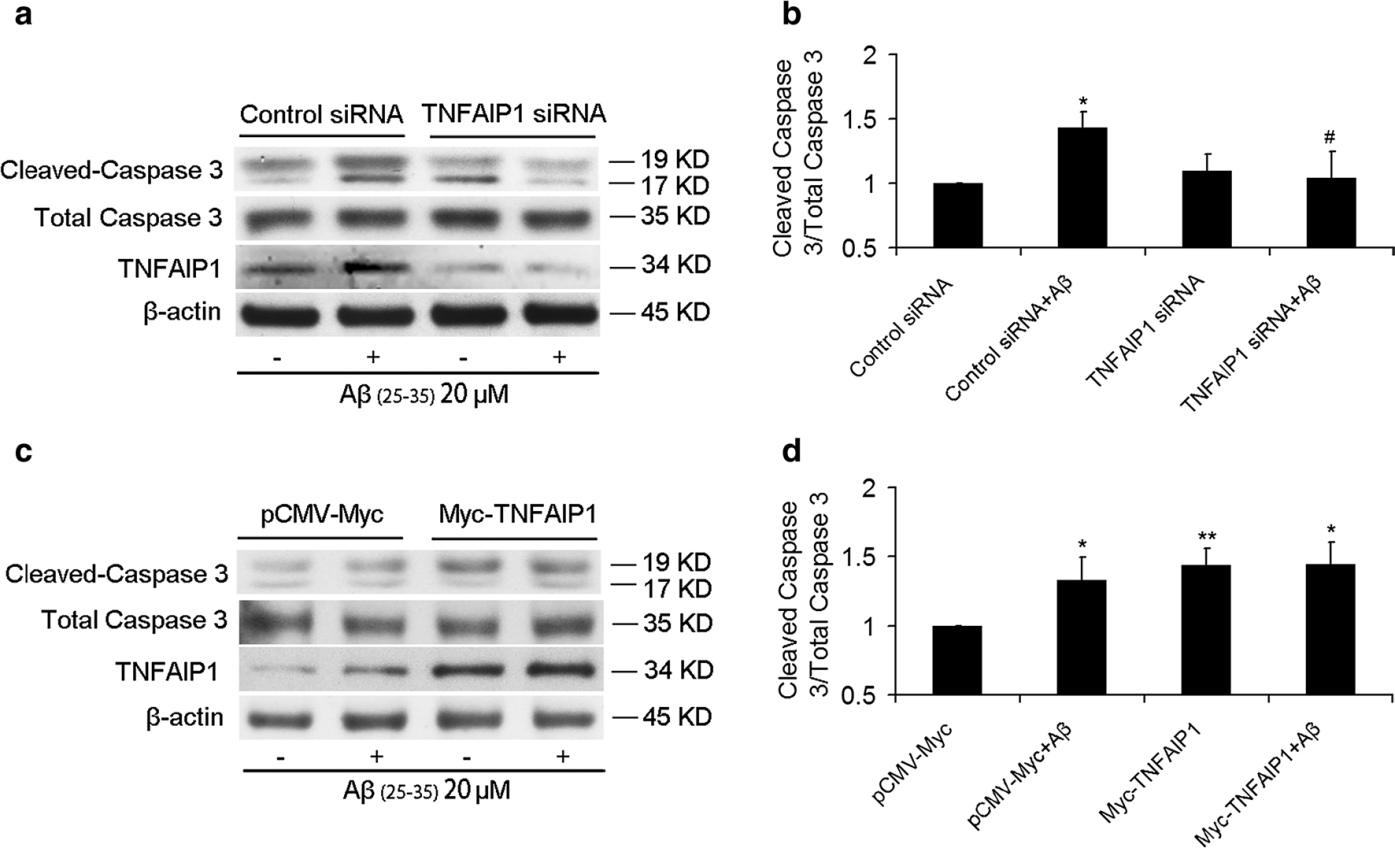
Effect of TNFAIP1 on Aβ_25–35_-induced the cleavage of caspase-3. **a** Cleaved caspase-3 protein levels were measured by Western blot in N2a cells cultured in 6 wells plate and transfected with 50 pmol of Control siRNA or TNFAIP1 siRNA, followed by treated by Aβ_25–35_ (20 μM). **b** Quantitative analysis of Cleaved caspase-3 protein levels (fold of control) in N2a cells. Data are expressed as mean ± SD, n = 3, *p < 0.05 compared with Control siRNA, ^#^p < 0.05 compared with Control siRNA plus Aβ_25–35_ (20 μM). **c** Cleaved caspase-3 protein levels were measured by Western blot in N2a cells cultured in 6 wells plate and transfected with 4 μg of pCMV-Myc or Myc-TNFAIP1, followed by treated by Aβ_25–35_ (20 μM). **d** Quantitative analysis of Cleaved caspase-3 protein levels (fold of control) in N2a cells. Data are expressed as mean ± SD, n = 3, *p < 0.05, **p < 0.01 compared with pCMV-Myc

Previous study suggested that TNFAIP1 is an apoptosis-related protein [12], implying that TNFAIP1 may also involve in Aβ_25–35_-induced apoptosis. Cleaved caspase-3 is a pivotal executioner and hallmark of apoptosis that is usually activated in the apoptotic cell by both extrinsic and intrinsic pathways. To further determine whether involvement of TNFAIP1 in Aβ_25–35_-induced neuronal apoptosis is associated with changes in the activities of caspases, cleaved caspase-3 was detected by Western blot. As shown in Fig. 3a, b, N2a cells treated with 20 μM of Aβ_25–35_ led to a significant increase in the level of cleaved caspase-3, while Aβ_25–35_-induced caspase-3 cleavage was significantly repressed by the knockdown of TNFAIP1. In contrast, 20 μM of Aβ_25–35_ treatment or Myc-TNFAIP1 transfection or Aβ_25–35_+Myc-TNFAIP1 co-treatment exhibited a significant increase in the cleavage of caspase-3 (Fig. 3c, d). These results indicate that TNFAIP1 contributes to Aβ_25–35_-induced apoptosis.

### TNFAIP1 inhibits Akt/CREB signaling pathway

**Fig. 4:**
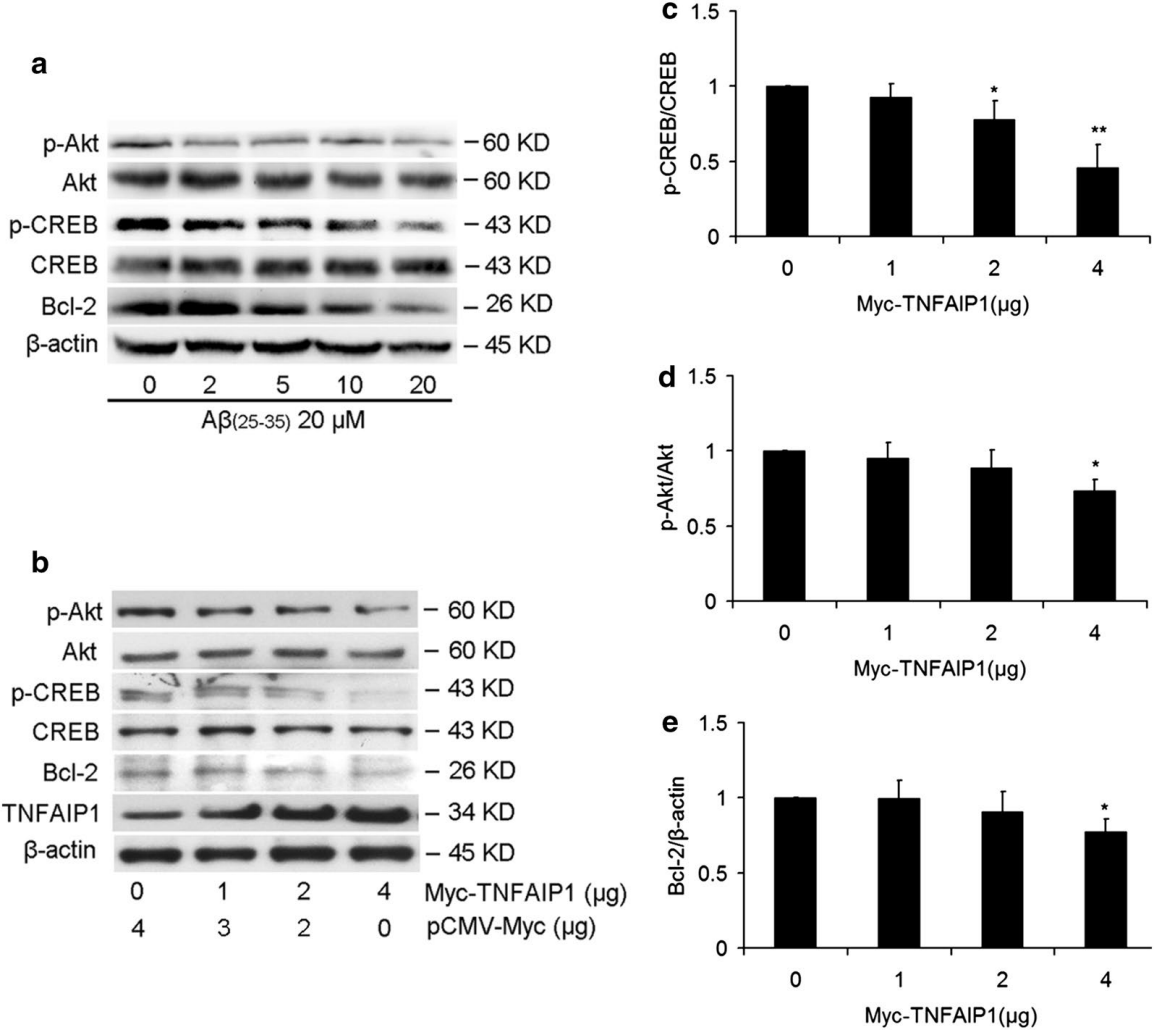
Effect of Aβ_25–35_ and TNFAIP1 on p-Akt, p-CREB and Bcl-2 expression. **a** p-Akt, p-CREB and Bcl-2 protein levels were measured by Western blot in N2a cells cultured in 6 wells plate and treated with dose-dependent Aβ_25–35_ (0–20 μM). **b** p-Akt, p-CREB and Bcl-2 protein levels were measured by Western blot in N2a cells cultured in 6 wells plate and transfected with Myc-TNFAIP1 and pCMV-Myc (0–4 μg). **c** Quantitative analysis of p-CREB protein levels (fold of control). Data are expressed as mean ± SD, n = 3, *p < 0.05, **p < 0.01 compared with Myc-TNFAIP1 (0 μg). **d** Quantitative analysis of p-Akt protein levels (fold of control). Data are expressed as mean ± SD, n = 3, *p < 0.05 compared with Myc-TNFAIP1 (0 μg). **e** Quantitative analysis of Bcl-2 protein levels (fold of control). Data are expressed as mean ± SD, n = 3, *p < 0.05 compared with Myc-TNFAIP1 (0 μg)

It has been reported that Akt/CREB signaling plays neuroprotective roles [18] and Aβ could downregulate Akt survival pathway [19]. In agreement with previous studies, our results showed that Aβ_25–35_ causes a decrease in Akt phosphorylation (phosphor-Ser473), CREB phosphorylation (phosphor-Ser133-CREB) and Bcl-2 expression in a dose-dependent manner (Fig. 4a). To further explore whether involvement of TNFAIP1 in neuronal apoptosis was associated with changes in Akt/CREB signaling pathway, we overexpressed TNFAIP1 in N2a cells in dose-dependent manner and then assessed Akt phosphorylation and CREB phosphorylation (Fig. 4a). As expected, overexpression of TNFAIP1 in N2a cells led to significant attenuation of p-Akt and p-CREB in a dose-dependent manner (Fig. 4b–d). Importantly, we also observed that overexpression of TNFAIP1 could significantly reduce the protein levels of Bcl-2 (Fig. 4e), which is an important neuroprotectant and transactivated by CREB in response to Aβ stimulation [20]. Therefore, these results indicate that TNFAIP1 may mediate Aβ_25–35_-induced apoptotic signaling by attenuating p-Akt, p-CREB and Bcl-2 expression.

### TNFAIP1 knockdown recovers Aβ_25–35_-induced CREB dephosphrylation and Bcl-2 downregulation

**Fig. 5:**
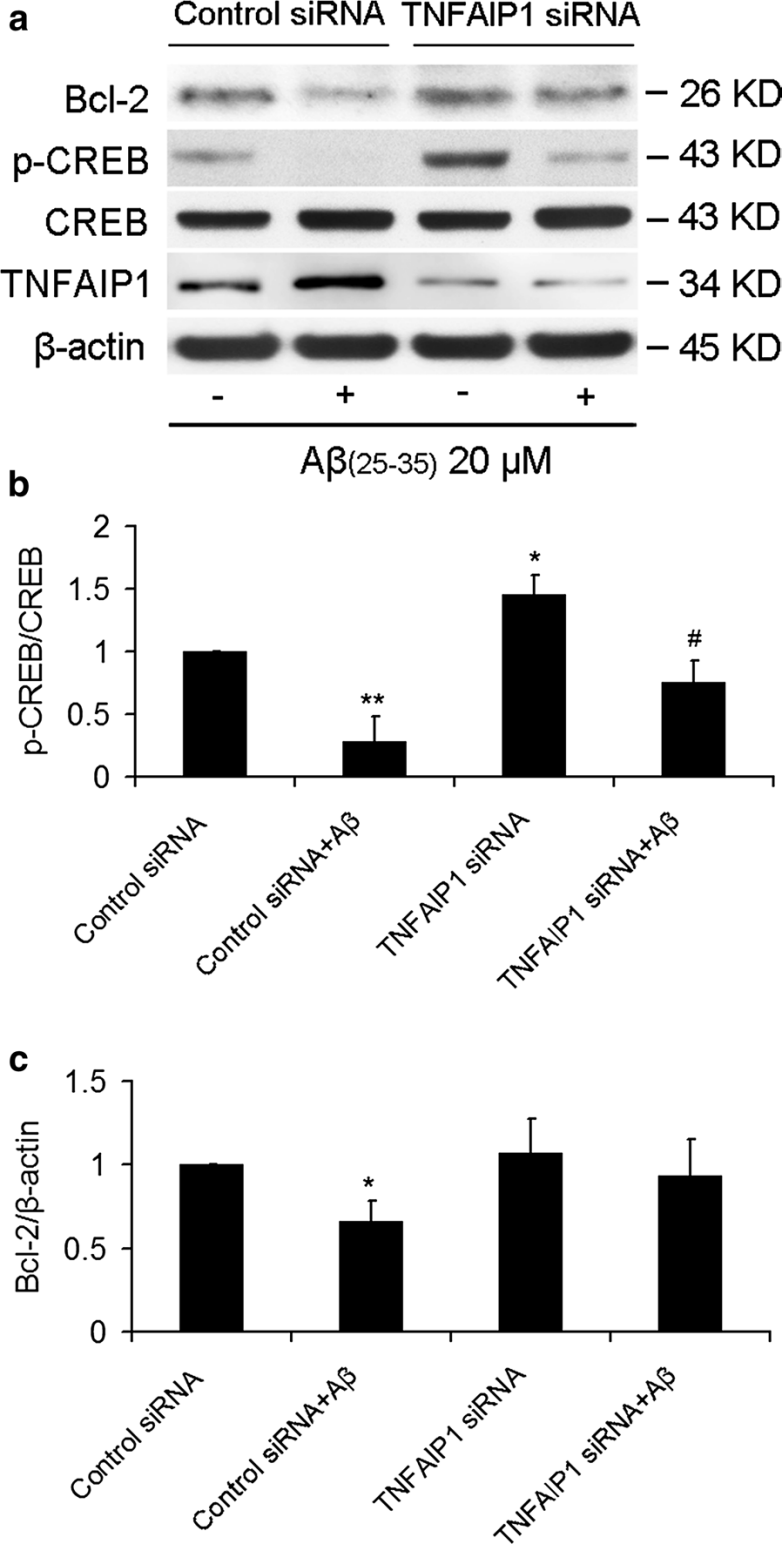
Effect of TNFAIP1 knockdown on Aβ_25–35_-decreased Bcl-2 and p-CREB. **a** Bcl-2 and p-CREB protein levels were measured by Western blot in N2a cells cultured in 6 wells plate and transfected with 50 pmol of Control siRNA or TNFAIP1 siRNA, followed by treated by Aβ_25–35_ (20 μM). **b** Quantitative analysis of p-CREB protein levels (fold of control). Data are expressed as mean ± SD, n = 3, *p < 0.05, **p < 0.01 compared with Control siRNA, ^#^p < 0.05 compared with Control siRNA plus Aβ_25-35_ (20 μM). (C). Quantitative analysis of Bcl-2 protein levels (fold of control). Data are expressed as mean ± SD, n = 3, *p < 0.05 compared with Control siRNA

Aβ had been reported to dramatically decrease CREB phosphorylation and Bcl-2 expression [20, 21]. To investigate the roles of TNFAIP1 in Aβ_25–35_-reduced CREB phosphrylation and Bcl-2 expression, control siRNA or TNFAIP1 siRNA was transfected into N2a cells for 24 h, followed by treatment with 20 μM of Aβ_25–35_. Consistent with previous studies [20], Aβ_25–35_ treatment significantly reduced CREB phosphrylation and Bcl-2 expression. TNFAIP1 siRNA but not control siRNA significantly upregulated CREB phosphorylation. However, TNFAIP1 siRNA could partially attenuate the inhibitory effect of Aβ_25–35_ on p-CREB and Bcl-2 (Fig. 5). These results indicated that inhibition of TNFAIP1 could attenuate Aβ-induced neurotoxicity partially by recovering the inhibitory effect of Aβ on p-CREB and Bcl-2 expression.

### TNFAIP1 contributes to Aβ_25–35_-induced CREB dephosphrylation and Bcl-2 downregulation

**Fig. 6:**
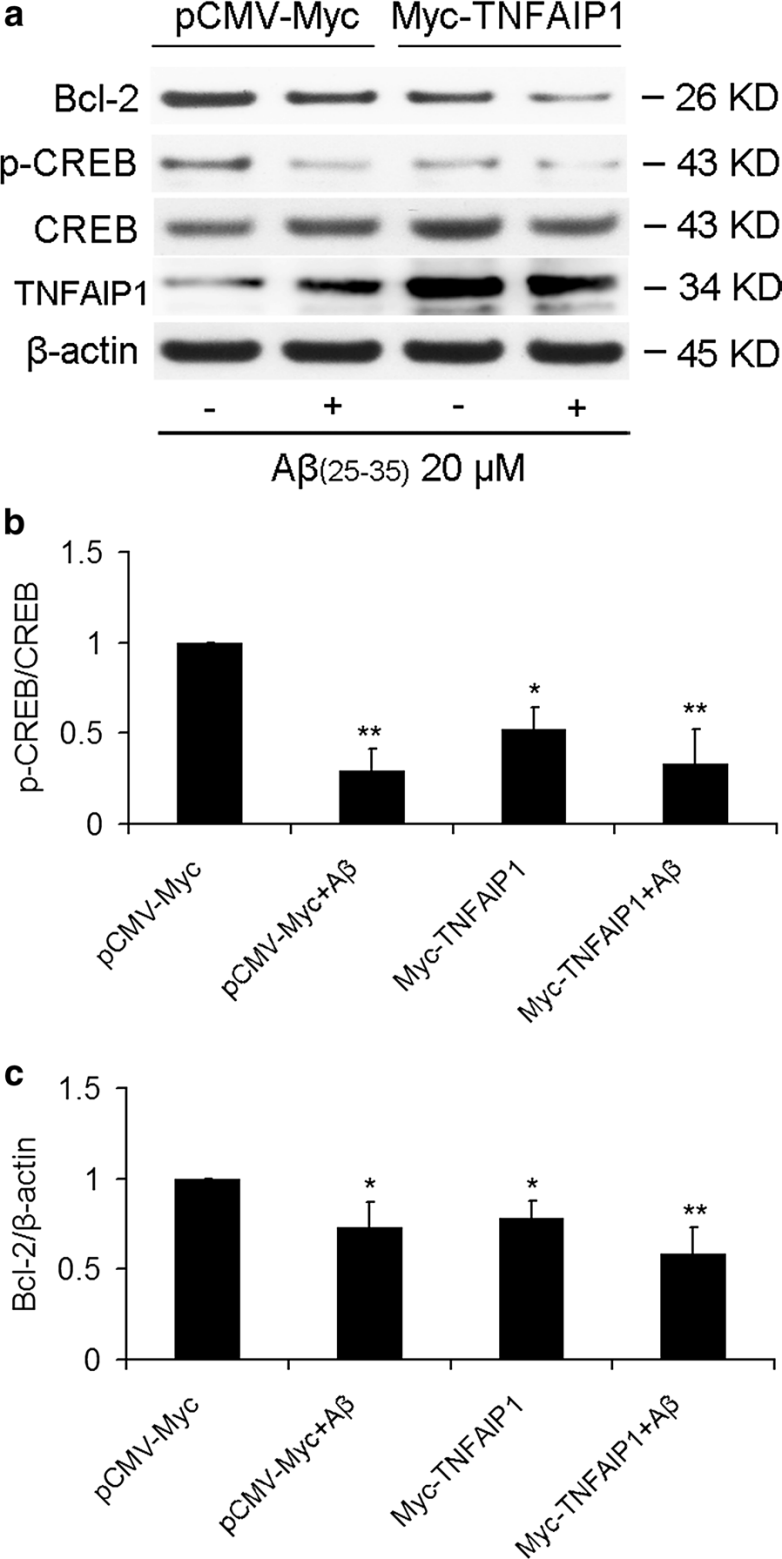
Effect of TNFAIP1 overexpression on Aβ_25–35_-decreased Bcl-2 and p-CREB. **a** Bcl-2 and p-CREB protein levels were measured by Western blot in N2a cells cultured in 6 wells plate and transfected with 4 μg of pCMV-Myc or Myc-TNFAIP1, followed by treated by Aβ_25–35_ (20 μM). **b** Quantitative analysis of p-CREB protein levels (fold of control). Data are expressed as mean ± SD, n = 3, *p < 0.05, **p < 0.01 compared with pCMV-Myc. **c** Quantitative analysis of Bcl-2 protein levels (fold of control). Data are expressed as mean ± SD, n = 3, *p < 0.05, **p < 0.01 compared with pCMV-Myc

The effect of TNFAIP1 overexpression on CREB phosphrylation and Bcl-2 expression under resting and Aβ_25–35_ treatment condition were also determined. Our results showed that 20 μM of Aβ_25–35_ or Aβ_25–35_+Myc-TNFAIP1 had exhibited almost equivalent inhibitory effect on the p-CREB (Fig. 6a, b). Moreover, we also observed that 20 μM of Aβ_25–35_ treatment, Myc-TNFAIP1 transfection, and Aβ_25–35_+Myc-TNFAIP1 co-treatment result in significant decrease in the expression of Bcl-2 (Fig. 6a, c). These results implied that TNFAIP1 maybe a key mediator involved in Aβ-induced neurotoxicity by inhibition of p-CREB and Bcl-2 expression.

## Discussion

Deposition of Aβ in the brain is a pathological hallmark of AD as it is responsible for progressive neurodegeneration in AD [22]. Accumulating evidences indicated that Aβ could cause neurotoxicity by inducing neuronal apoptosis in vitro and in vivo [23, 24]. Elucidating the molecular mechanisms underlying Aβ-induced neuronal apoptosis will help us on developing treatment of AD. However, despite intense research, it remains unclear how Aβ triggers the signaling cascade that results in neuronal dysfunction and neurotoxicity. In the present study, our results demonstrated that TNFAIP1 protein is induced by Aβ_25–35_ and involved in Aβ_25–35_-induced neuronal apoptotic pathway because overexpression of TNFAIP1 can accelerate Aβ_25–35_-induced apoptosis while inhibition of TNFAIP1 can significantly reduce Aβ_25–35_-induced neuronal apoptosis. Moreover, we found that TNFAIP1 was involved in Aβ_25–35_-induced neuronal apoptosis by deactivating Akt signaling pathway and CREB transcriptional activity.

The transcript levels of TNFAIP1 was found to be robustly induced in the transgenic *C. elegans* AD brains and post-mortem AD brain in previous study [12, 13], which led us to examine the hypothesis that TNFAIP1 was involved in the pathological development of AD using an in vitro mouse AD model: mouse primary cortical neurons and N2a neuroblastoma cells treated by Aβ_25–35_. Our results suggested that Aβ_25–35_ could induce TNFAIP1 protein and mRNA levels in a dose-dependent manner. Furthermore, overexpression or knockdown of TNFAIP1 exacerbated or alleviated Aβ_25–35_-induced neurotoxicity. Together, these results indicated that the TNFAIP1 gene expression may be critical for Aβ_25–35_-induced neuronal death and intervention of its expression could be a potential method for inhibiting Aβ_25–35_-induced neurotoxicity. However, the regulatory mechanisms of TNFAIP1 remain largely unclear. Only a recent report described that transcription factor Sp1 could bind to human *TNFAIP1* promoter region and transactivate *TNFAIP1* promoter [25]. As TNFAIP1 had been originally found to be induced by TNFα [9], which is a potent activator of NFκB, thus it is possible that TNFAIP1 was also regulated by NFκB when exposed to Aβ_25–35_. Further studies will be performed to elucidate whether inhibition of NFκB activation can reduce Aβ_25–35_-induced *TNFAIP1* gene expression.

Previous studies described that TNFAIP1 was a pro-apoptotic protein [12], and apoptosis is a general neuronal death pathway in neurodegenerative diseases which could be triggered by exposure to Aβ. In the present study, we extended our observations to in vitro AD models that TNFAIP1 was also involved in Aβ_25–35_-induced apoptosis. We found that inhibition of *TNFAIP1* gene by specific siRNA significantly attenuated Aβ_25–35_ induced cleaved-caspase 3. As caspase-3 cleavage is a central event in executing Aβ_25–35_-induced neuronal apoptosis [26, 27], these results provide further evidence that TNFAIP1 was involved in Aβ_25–35_-induced apoptosis. However, it is worth noting that Aβ_25–35_+Myc-TNFAIP1 co-treatment did not cause a much higher increase in the cleavage of caspase-3 than Aβ_25–35_ treatment or Myc-TNFAIP1 transfection alone. It is possible that 20 μM of Aβ_25–35_ treatment or Myc-TNFAIP1 transfection alone had already exerted a relatively strong apoptotic effect on N2a cells, which led to a insignificant effect on caspase 3 cleavage by additional other stimuli. This issue will be further defined in future studies. Thus, these results are in support of the hypothesis that the induction of TNFAIP1 expression is part of the intrinsic program of apoptotic neuronal death induced by accumulation of Aβ in AD.

It had been demonstrated that CREB is directly phosphorylated and regulated by the protein kinase Akt [28], and Akt/CREB signal has been shown to play a pivotal role in neuroprotection by enhancing cell survival and inhibiting apoptosis [18]. Importantly, Akt and CREB could promote neuronal cell survival by upregulating Bcl-2 protein levels [29]. As expected, TNFAIP1 could reduce p-Akt, p-CREB and Bcl-2 protein levels in a dose-dependent manner. Moreover, we observed overexpression of TNFAIP1 could further attenuate Aβ-inhibited p-CREB and Bcl-2 protein expression, whereas inhibition of TNFAIP1 could significantly reverse the effect of Aβ on p-CREB and Bcl-2. These results suggest that TNFAIP1 may mediate the deactivation of CREB induced by Aβ, and then trigger neuronal apoptosis. MAPKs had been reported to be involved in Aβ-induced apoptosis, and MAPKs signaling cascades were activated in brains from AD patients [30]. Moreover, Aβ-induced MAPKs include ERK1/2, JNK and p38 MAPK had been demonstrated to be associated with neuronal cell death [31, 32]. Therefore, TNFAIP1 may also modulate MAPKs signaling cascades involved in Aβ-induced neuronal apoptosis. Further study will be conducted to elucidate the role of TNFAIP1 in modulating MAPKs signaling pathway involved in the apoptotic effect of Aβ.

In summary, in this study we found that TNFAIP1 expression was elevated in mouse primary cortical neurons and N2a cells exposed to Aβ_25–35_. TNFAIP1 overexpression was correlated with neuronal apoptosis initiated by Aβ_25–35_ and inhibition of TNFAIP1 was able to reduce the neuronal damage induced by Aβ_25–35_ in vitro AD models. Moreover, our results suggested that TNFAIP1 may mediate Aβ_25–35_-induced neuronal damage by inhibiting p-CREB and Bcl-2 protein levels. Our results implied that inhibition of *TNFAIP1* gene during AD process could be a potential therapeutic strategy for treatment of AD. Further studies will be performed to determine the roles of TNFAIP1 in in vivo AD models.

## Conclusions

In summary, this study clearly demonstrated that TNFAIP1 was significantly upregulated by Aβ_25–35_ in mouse primary cortical neurons and N2a cells, and TNFAIP1 may be a key player that mediated Aβ_25–35_-induced neurotoxicity by inactivating the Akt/CREB signaling pathway, and in turn downregulating anti-apoptotic protein Bcl-2. Our findings implied that TNFAIP1 may be a potential therapeutic target for treatment of AD, but the roles and mechanism of TNFAIP1 in in vivo AD models need to be further elucidated in our future study.

## Abbreviations

Aβ: amyloid-beta
AD: Alzheimer’s disease
N2a: Neuro2a
TNFAIP1: tumor necrosis factor, alpha-induced protein 1
TNFα: tumor necrosis factor alpha
APP: β-amyloid precursor protein
PCNA: proliferating cell nuclear antigen
ATCC: The American type culture collection
RT-PCR: real-time PCR
